# Brief Pollination Assessment of a Critically Endangered Food-Deceptive Orchid (*Cypripedium guttatum*) Using a Network Approach

**DOI:** 10.3390/plants11060798

**Published:** 2022-03-17

**Authors:** Hakbong Lee, Heung-Sik Lee, Kee-Hwa Bae

**Affiliations:** 1 Korea National Arboretum, Pocheon 11186, Korea; ppnet113@nie.re.kr; 2 Center for Endangered Species Restoration, National Institute of Ecology, Seocheon-gun 36531, Korea; 3 Animal and Plant Quarantine Agency, Gimcheon 39660, Korea; lhsgo@korea.kr; 4 Seed Vault Center, Baekdudaegan National Arboretum, Bonghwa-gun 36209, Korea

**Keywords:** ecological context, effective pollinator, nestedness, network, slipper orchid

## Abstract

The translocation of orchids (Orchidaceae) cannot be successful if one is unaware of their effective pollinators and plant–pollinator interactions. *Cypripedium guttatum* is a generalized food-deceptive orchid, which is highly threatened in the Republic of Korea, thus, requiring immediate translocation actions. Although effective pollinators of the orchid are well known in China, little is known about the pollinators in the Republic of Korea and the ecological context in which the orchid can be successfully pollinated. To briefly assess the pollination of *C. guttatum* prior to translocation, we conducted a one-month survey of general pollination and the community-wide plant–pollinator network properties. Over 21 h of observation, we found that an effective pollinator of the orchid was the sweat bee *Lasioglossum virideglaucum*. The network was significantly specialized and modular, but not significantly nested. *L. virideglaucum* (pollinator) and *Arabis gemmifera* (plant) were determined to be keystone species, based on network metrics. A total of six network modules were identified and the flower colors of the plant species belonging to the *C. guttatum* module were purple, white, and yellow. After comparing the daily network patterns, we found that pollination of the orchid was accomplished when various flowering plant species bloom, and the nestedness value was high. This study revealed that high plant and pollinator richness could increase the chance that the deceptive orchid would be pollinated. Our study suggests that the network properties of this food-deceptive orchid community could provide useful insight into understanding the ecologically suitable habitat for the translocation of the highly threatened orchid species *C. guttatum*.

## 1. Introduction

Orchids (Orchidaceae) are the world’s second-largest family of flowering plants (c. 28,000) [1]. They are adapted to diverse ecosystems but are highly threatened plants. Recently, The International Union for Conservation of Nature (IUCN) reported that 1636 orchid species were assessed as threatened, five species were extinct, and the state of the other species (c. 94%) remained unknown [2]. The global orchid decline is mainly attributed to extrinsic factors, such as habitat destruction and fragmentation, over-collecting, and climate change [3,4,5]. Further, intrinsic factors (e.g., life-history) have been recognized by orchid biologists as critical for the decline [4,6]. Thus, conservation actions to reduce the extinction risk are urgently needed.

Translocation is widely used as a conservation tool to ameliorate the known threats to orchid species [4,7,8]. However, without understanding the key ecological attributes of the orchids (e.g., relationship with pollinators or mycorrhizal fungi), translocation actions cannot be successful [4]. Indeed, in many cases of orchid translocations, the pollinator presence at the translocated sites was rarely determined, which could have revealed low recruitment rates to the translocated sites [4]. In addition, plant–pollinator interactions, between the target species and recipient community, should be fully understood before applying any conservation translocation measures (e.g., reintroduction and reinforcement) [9] to orchid species [4,6,7,10].

Slipper orchids (genus *Cypripedium*) are one of the most intensively studied orchid groups due to their unique life history traits, such as pollination, and most of them (ca. 90% of species in subfamily Cypripedioideae) were assessed as threatened [11]. *Cypripedium* species are non-rewarding orchids and pollinated by deceit, mainly as food mimics, and rarely as nesting-site mimics or brood-site mimics [12]. Usually, in *Cypripedium* species, pollination is accomplished by insect pollinators falling into the pouch-like labellum, escaping through a posterior opening, and visiting another flower of the same species. The pollinators of *Cypripedium* species are bees, flies, and occasionally, wasps [12,13,14]. Among the pollinators, bees account for most of their effective visitations [12,15].

To conserve *Cypripedium* species, understanding the ecology of co-occurring rewarding plants in the community is a prerequisite, although identifying the effective pollinators is important. These orchids employ generalized food deception strategies [16,17], and such food-deceptive orchids display general floral signals (e.g., visual or olfactory) that attract various insect visitors [18,19]. Further, the spectrum of foraging pollinators visiting *Cypripedium* species is generally wide, as they visit various flowers for food (i.e., polylectic) [20,21,22,23,24,25]. Thus, the pollination of *Cypripedium* species is likely to be facilitated when rewarding plants around their populations are blooming sufficiently to feed diverse pollinators [26,27]. In this case, the understanding of community-wide plant–pollinator interactions is needed to identify co-occurring plants that function to support the populations of potential orchid pollinators [28]. However, pollination studies on deceptive orchids have rarely attempted to identify effective pollinators or examine plant–pollinator interactions from community perspectives [21,29].

Understanding the structure and dynamics of the interactions between plants and pollinators can provide new insight into the ecological context, in which this deceptive orchid is effectively pollinated. In real-world ecosystems, species are interlinked with each other [30]. Thus, for species conservation, we cannot consider only the pairs of interacting species [31,32], as have many previous studies, which focused on the interactions between the target orchid species and their effective pollinators. Network analysis is a tool that has been employed for understanding the structure and dynamics of plant–pollinator interactions, as well as identifying topologically important species within the networks [33,34,35]. For example, metrics at the network level (e.g., nestedness, specialization, and modularity) are used in describing network structure and provide information on structural stability, resilience, and fragility [33,36,37]. At the species level, the unweighted degree (i.e., number of interaction partners) [38] and centrality (i.e., centrality and closeness centrality betweenness) [35] have been frequently used to determine the topological importance of each species (e.g., keystone species), which can be achieved with low sampling effort [39]. Because a pollination network survey generally involves observing all flowering plants and their visitors within a community and detecting changes in those interactions over time, community-wide plant–pollinator interactions can be more clearly understood than pollination surveys at the population level. In addition, the network approach has received growing attention as a tool for understanding the ecological context, since a robust method for comparing networks with different sizes was developed [40]. However, the network approach has rarely been applied in studies regarding the pollination of deceptive orchids [41].

*Cypripedium guttatum* is a critically endangered slipper orchid, especially in the Republic of Korea, although the orchid is one of the most widely distributed slipper orchids in the world [21]. In the Republic of Korea, only two isolated populations remain, and thus, translocation action is urgently needed. Pollinators (e.g., Halictid bees; *Lasioglossum* spp.) visiting *C. guttatum* and their interactions with rewarding plants have been comprehensively studied in China [21]. However, there is no such information for the populations in the Republic of Korea, hindering effective conservation actions for the orchid.

In the present study, we aimed to briefly assess the pollination of the slipper orchid *C. guttatum*, thus, providing useful insight into the successful translocation of the orchid. To this end, we examined flowering phenology, the effective pollinators of *C. guttatum*, and the structure and dynamics of community-wide plant–pollinator interactions by applying a network approach.

## 2. Materials and Methods

### 2.1. Study Site

**Figure 1:**
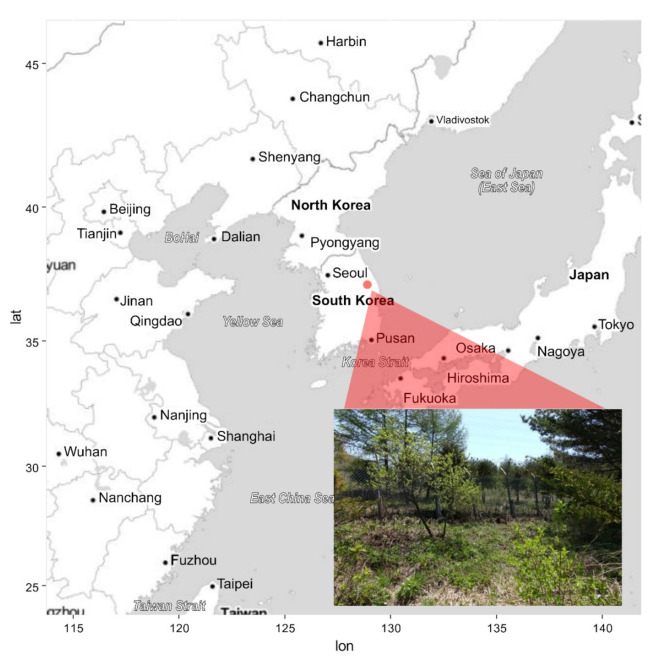
Habitat location of *Cypripedium guttatum* in the Republic of Korea. The red point is Manhangjae where *C. guttatum* is located. The photograph was taken within the artificial fence (lat = latitude, lon = longitude).

In the Gangwon Province in the Republic of Korea, *C. guttatum* is distributed at only two sites (Manhangjae and Jeongamsa). The present study was conducted at the Manhangjae site (37°08′57.3″ N, 128°54′10.8″ E; altitude 1 271 m a.s.l.; Figure 1). This species is classified as critically endangered according to the Wildlife Protection and Management Act legislated by the Ministry of Environment in the Republic of Korea, and for this reason, a rectangular fence is installed around the habitat to prevent illegal trespassing. The site is situated on the northwestern slope (326°) and mainly consists of trees (*Larix kaempferi*, *Pinus densiflora*, *Pinus koraiensis*, and *Quercus mongolica*; height > 7 m) and shrubs (*Fraxinus rhynchophylla*, *Quercus mongolica*, *Salix caprea*, *Tripterygium regelii*, and *Weigela florida*). The mean air temperature of the site during the study period (12 May to 7 June 2018) was 14.9 ± 6.0 °C (Hobo UA-002-64; Onset Comp. Corp., Bourne, MA, USA).

### 2.2. Flowering Phenology

In 2018, a total of 49 *Cypripedium* shoots were observed at the Manhangjae site. Only four of the observed shoots developed flower buds, and the flowering of these shoots was observed every 2 d on average (range, 1–4 d) for a total of 15 observations until the flowers wilted (from 12 May to 7 June 2018). The flowering duration of each flower was also measured. Because the pollinators were only able to access *C. guttatum* flowers when a gap between the upper sepal and the labellum opened, flowering duration was defined as the number of days between the opening and closing of the gap.

### 2.3. Effective Pollinators

Pollinator observations were performed three times during the flowering period, coinciding with early, mid, and late flowering (28, 31 May and 4 June 2018, respectively). The observations were made from 0900 to 1600 h for a total of 7 h per day. The insects visiting the four flowering shoots were recorded, and effective pollinators were defined as those that entered the labellum of a flower, escaped with pollen loads, and subsequently visited conspecific flowers according to Argue [42]. The visitation frequency and visitation duration of the effective pollinators were also recorded, where the visitation duration was defined as the period of time between entering and escaping the labellum (*N* = 8). The insects that only roamed or landed on the upper sepal or the labellum were excluded from the visitation frequency observations. Both the observed effective pollinators that escaped the labellum in a legitimate way, as well as the ineffective visitors, were collected into a killing jar that contained ethyl acetate. Among them, hymenopterans were identified to the species level by a bee expert (H.-S.L.).

### 2.4. Network Sampling

A quadrant (40 × 40 m) that was large enough to provide a full representation of the floristic composition of the study site was installed around *C. guttatum* at the Manhangjae site. Network sampling was conducted during both the pre-flowering (26 May 2018) and anthesis stages of *C. guttatum* (27, 29 May and 3 June 2018). During the survey period, 12 insect-pollinated plants including *C. guttatum* were found (Table 1). Because *C. guttatum* is known to be rarely visited by pollinators [21] and it is quite rare at this site, a timed observation method was used. Such an approach is more appropriate for the study of rare species and is likely to reveal rarer interactions than the transect method [43]. A daily sampling schedule was established with three time slots, mainly in the morning (0900–1200 h), early afternoon (1200–1500 h), and late afternoon (1500–1800 h), with each plant species being allocated an equal amount of observation time according to Carvalheiro et al. [44]. Because the structure of the flowers varies among plant species, we defined the observation unit for each species according to its flower structure. If a species had one or two flowers (e.g., *Polygonatum odoratum* var. *pluriflorum*, *Ranunculus japonicus*, *Viola mandshurica*, *Rhododendron schlippenbachii*, and *C. guttatum*), then the observation unit was one or two flowers. If flowers were in inflorescences (e.g., *Aruncus dioicus* var. *kamtschaticus*, *Weigela florida*, *Arabis gemmifera*, *Taraxacum officinale*, *Barbarea vulgaris*, *Valeriana fauriei*, and *Cerastium holosteoides* var. *hallaisanense*), then the observation unit was one or two inflorescences. We spent 10 min observing each plant species and recorded the number of visits when an insect contacted a stigma or anther. The effective pollinators were identified at the species, genus, or family level.

### 2.5. Network Analysis

We calculated widely used network analysis metrics (connectance, weighted nestedness, complementary specialization, and quantitative modularity) to characterize the plant–pollinator network structure of the *C. guttatum* community. A quantitative network matrix was constructed using visitation frequency (i.e., number of visits). Connectance (C) was calculated as the ratio of the observed interactions to possible interactions. Nestedness (i.e., nestedness based on overlap and decreasing fill, NODF) [45] indicates the tendency of specialist species to interact with subsets of the species that interact with generalist species in a network, with greater values (range, 0–100) indicating stronger tendencies. Complementary specialization (H_2_′) is a measure of the degree to which a network is specialized, and ranges from 0 (complete generalization) to 1 (complete specialization) [36]. Quantitative modularity (Q) was calculated using the QuanBiMo algorithm [46] to identify the groups that strongly interacted with each other (i.e., modules). The Q value ranges from 0 to 1, with higher values indicating greater degrees of compartmentalization (i.e., a lack of interaction between modules). Because the Q value changes during every run due to the Markov chain Monte Carlo move, the iteration with maximum likelihood was selected after running the model for 100 repetitions. Because network indices *per se* cannot determine statistical significance, 1000 randomized networks were generated using Patefield’s algorithm (i.e., having the same marginal totals as the observed network) [47]. We compared the network level metrics (NODF, H_2_′, and Q) from the random networks to those from the observed network to determine statistical significance (95% confidence interval (CI)). To determine the topological roles of plants and pollinators in the investigated network, we calculated the unweighted degree (i.e., the number of interaction partners for each species) and centrality scores, including betweenness centrality (BC) and closeness centrality (CC) [35]. In mutualistic networks, keystone species generally yield high BC and CC values [35]. We additionally presented the temporal dynamics of the networks and compared nestedness among the daily networks using Song’s approach (i.e., combined NODF) [40]. Combined NODF (NODF_c_) is a reliable measure to compare nestedness across networks with different sizes and connectance [40]. All network metrics were calculated using the bipartite package (ver. 2.16) [48] in R (ver. 4.0.4) [49], and the maxnodf package [50] was used for nestedness comparisons between the daily networks.

## 3. Results

### 3.1. Flowering Phenology

The flowering period of *C. guttatum* in the Manhangjae site lasted from 27 May 2018 (Julian day, 147) to 6 June 2018 (157), for a total of 10 d. The four flowering shoots did not differ in the time of flowering onset or in the duration of flowering. Just before the end of the flowering period, the upper sepals of the flowers wilted, after which the labellum contorted and the structural form that allowed pollinator access was lost.

### 3.2. Effective Pollinators

**Figure 2:**
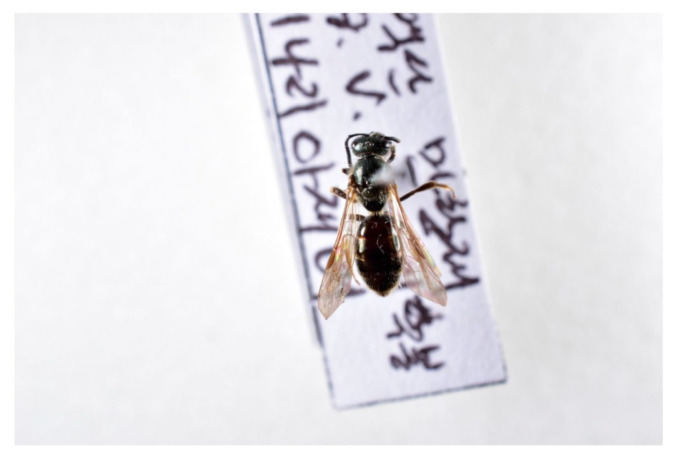
The effective pollinator (*Lasioglossum virideglaucum*) of *Cypripedium guttatum* at the Manhangjae site in the Republic of Korea. The photograph was taken by H.-S.L., and the specimen was deposited in the Plant Quarantine Technology Center in the Republic of Korea.

**Figure 3:**
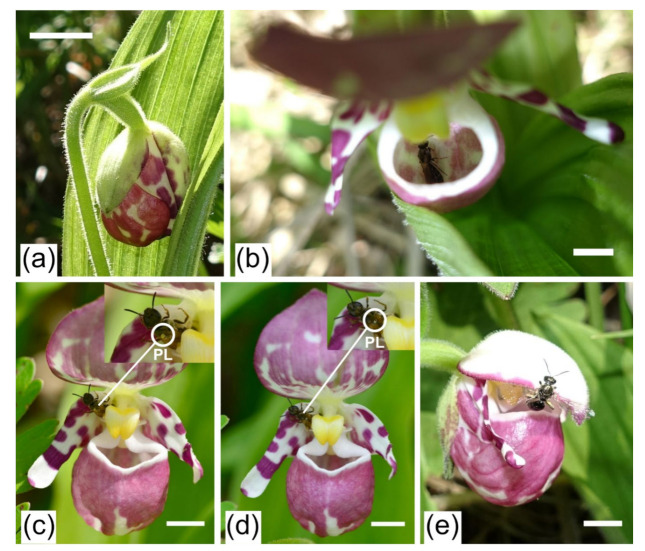
Flower and effective pollinators of *Cypripedium guttatum* (**a**). Large flower bud (scale bar = 10 mm). (**b**). A sweat bee (*Lasioglossum virideglaucum*) trapped in the flower (scale bar = 5 mm). (**c**), (**d**). When *L. virideglaucum* escaped the flower, its upper thorax was smeared with large (C) or small (D) pollen loads, which could be transported to the stigma of the other flowers (scale bar = 5 mm). (**e**). *Lasioglossum miyabei* escaped the flower by crawling along the upper surface of the sepal with no pollen load (PL = pollen load, scale bar = 5 mm).

A false blister beetle (*Oedemeridae* sp. 1), two sweat bees (*Lasioglossum virideglaucum* and *L*. *miyabei*), and a drone fly (*Eristalomyia tenax*) were observed to visit the flowers of *C. guttatum*. Only *L*. *virideglaucum* (♀; Figure 2) legitimately visited the flower, whereas the other three visiting species landed either on the upper sepal or the lateral petal and did not fall into the labellum. The *L*. *virideglaucum* individuals fell into the labellum, crawled to the posterior opening, and finally, escaped the flower with pollen loads smeared on the upper side of their thorax (Figure 3). Such effective visitation took an average of 62.6 ± 10.4 s (mean ± SD, range, 45–78 s, *N* = 8; Table 2). After escaping a flower, these pollinators consecutively visited conspecific flowers. During the flowering period of *C. guttatum* (from 28 May to 4 June 2018), the majority of the visits by *L*. *virideglaucum* occurred between 1400 h and 1500 h (Table 3).

### 3.3. Plant–Pollinator Network

**Figure 4:**
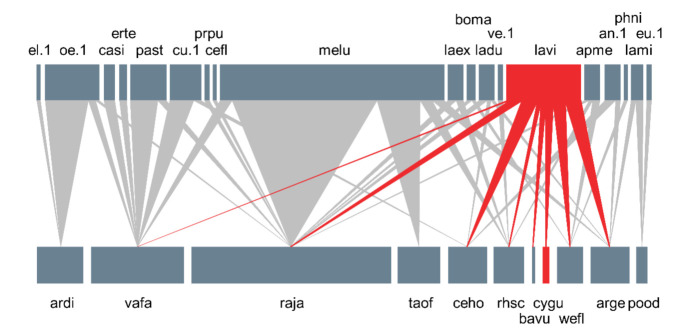
Plant–pollinator network in the natural habitat of the critically endangered orchid *Cypripedium guttatum*. The upper and lower boxes indicate pollinators and plants, respectively. The width of each box and vertical line are proportional to the interaction frequency. The red upper box and vertical lines indicate interaction diversity and the abundance of *Lassioglossum virideglaucum*, and the lower red box represents *C. guttatum* (pollinator: an. 1 = Andrena sp. 1, apme = *Apis mellifera*, boma = *Bombylius major*, casi = *Carterocephalus silvicola*, cefl = *Ceratina flavipes*, cu. 1 = *Curculionidae* sp. 1, el. 1 = *Elateridae* sp. 1, erte = *Eristalomyia tenax*, eu. 1 = *Eucera* sp. 1, laex = Lasioglossum exiliceps, ladu = *Lasioglossum duplex*, lavi = *Lasioglossum virideglaucum*, lami = *Lasioglossum miyabei*, melu = *Metasyrphus luniger*, oe. 1 = *Oedemeridae* sp. 1, past = *Parnassius stubbendorfii*, phni = *Philopota nigroaenea*, prpu = *Pristomyrmex pungens*, ve. 1 = *Vespidae* sp. 1; Plant: arge = *Arabis gemmifera*, ardi = *Aruncus dioicus* var. *kamtschaticus*, bavu = *Barbarea vulgaris*, ceho = *Cerastium holosteoides* var. *hallaisanense*, cygu = *Cypripedium guttatum*, pood = *Polygonatum odoratum* var. *pluriflorum*, raja = *Ranunculus japonicus*, rhsc = *Rhododendron schlippenbachii*, taof = *Taraxacum officinale*, vafa = *Valeriana fauriei*, wefl = *Weigela florida*).

A total of 31 species, including 12 insect-pollinated plant species and 19 pollinator species, was observed at the Manhangjae site, with a total of 42 interactions (Figure 4). On average, each species interacted with 1.4 other species. Network connectance (C) was calculated as 0.201, and the network was not significantly nested compared to randomized networks (NODF = 18.849, 95% CI: 27.663–28.061). Meanwhile, the specialization level (H_2_′) of the network (0.507) was significantly higher than that of the randomized networks (95% CI: 0.142–0.145), and the network was also significantly modular (Q = 0.510, 95% CI: 0.209–0.216).

A syrphid fly (*Metasyrphus luniger*) accounted for almost half (41.0%) of the total interaction frequencies and visited 5 of the 12 plant species (unweighted degree = 5; Figure 4). In contrast, *L*. *virideglaucum* was less dominant in terms of interaction frequency (13.7%), but it was observed to visit a greater number of plant species (8 out of 12; Figure 4). Among the eight species visited by *L*. *virideglaucum*, about three-quarters of the visits (73.8%) were made to *A*. *gemmifera* (21.1%), *C*. *holosteoides* var. *hallaisanense* (21.1%), *R*. *japonicus* (15.8%), and *W*. *florida* (15.8%; Figure 4).

**Figure 5:**
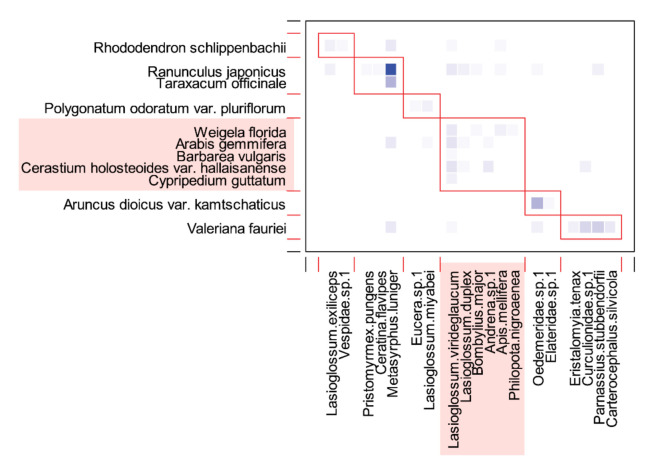
Modules identified by the QuanBiMo algorithm in the *Cypripedium guttatum* community of the Manhangjae habitat. Plants and pollinators are arranged in rows and columns, respectively. The boxes with red border lines indicate the modules, which total six. Each square with a color gradient indicates the interaction strength, with darker colors representing more frequent interactions. Component species in the module to which *C. guttatum* belongs are represented as rectangular boxes, colored pale red.

A total of six network modules were identified (Figure 5), and *C. guttatum* belonged to the largest module (i.e., contained the most species), along with plants that had purple (*W*. *florida* and *C. guttatum*), white (*A*. *gemmifera* and *C*. *holosteoides* var. *hallaisanensis*), or yellow (*B*. *vulgaris*) flowers. All plants within the *C. guttatum* module had between one and five pollinator partners, except for *R*. *japonicus*, which interacted with 10 pollinators. Bees accounted for a high proportion of the constituent species in the *C. guttatum* module (four of six species), whereas flies accounted for a lower proportion (two of six species; Figure 5).

We identified the keystone pollinator in the network using unweighted degree and centrality scores (BC and CC) and, surprisingly, the only effective pollinator of *C. guttatum*, *L. virideglaucum* was identified as a keystone pollinator (Table 4). *A*. *gemmifera* was identified as a keystone plant with the highest centrality scores among the plant species (Table 5).

**Figure 6:**
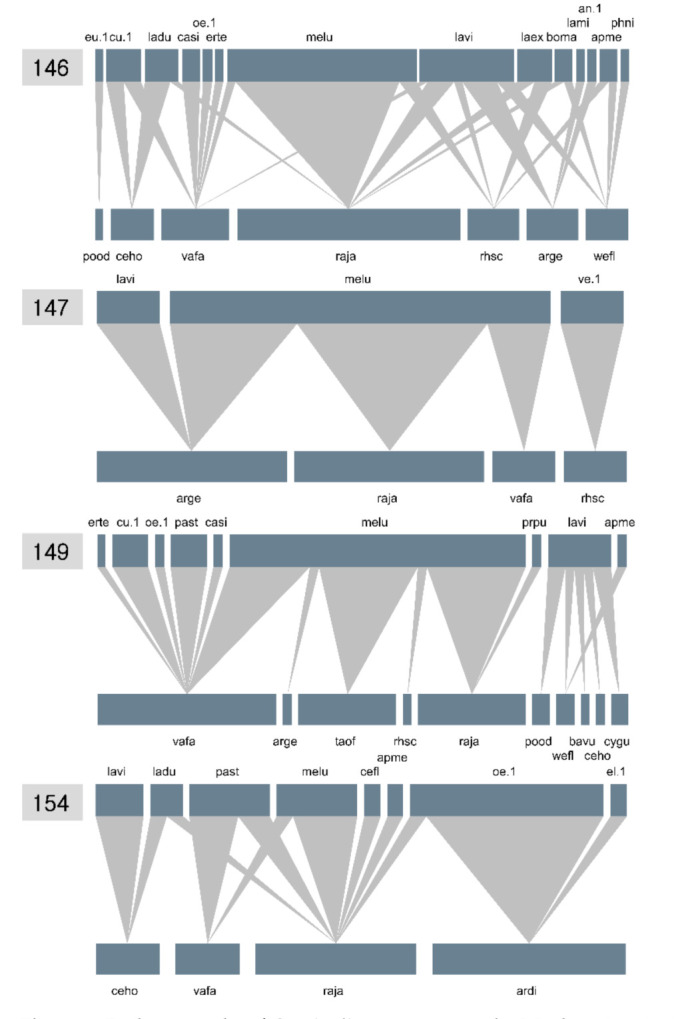
Daily networks of *Cypripedium guttatum* at the Manhangjae site in the Republic of Korea. The numbers on the upper-left side of each network indicate the days of the year. The acronyms for each network are shown in Figure 4.

In the daily networks, the number of plant species with fully opened flowers fluctuated greatly over the period of the network survey (9 days, 7→4→10→4; Figure 6). *L*. *virideglaucum* visited more diverse flowers on days 146 and 149, when the number of species with fully opened flowers was high. In contrast, the visitation degree (i.e., the number of partner plants) was the lowest when the number of flowering plants was the lowest (days 147 and 154; Figure 6). Using combined NODF, we compared nestedness among the daily networks. During the pre-flowering (day 146) and anthesis stages (147, 149, and 154) of *C. guttatum*, an effective pollinator of *L*. *virideglaucum* visited the orchid on day 149 (Figure 6), and the NODF_c_ value on that day was the highest among the four survey dates (147, 149, and 154) (Table 6).

## 4. Discussion

Assessing pollination, a key ecological attribute, is essential for the conservation of critically endangered Slipper orchids (e.g., *C. guttatum*). Because *Cypripedium* species are mostly highly interlinked with co-occurring species, the examination of plant–pollinator interactions from network perspectives can provide new insight into the translocation of the orchids. We demonstrated that *C. guttatum* in the Republic of Korea was only pollinated by *L*. *virideglaucum* over the observation period, and the effective pollinator was determined as a high-priority keystone species for community conservation, in terms of the maintenance of the target orchid and co-occurring species. Given that the identification of a keystone plant or pollinator in a network can be achieved with low sampling effort [39], well-connected species (i.e., keystone pollinator) persist over time [34]. Since *C. guttatum* and *L*. *virideglaucum* were in the same module, the pollination of *C. guttatum* is likely to be maintained over time. Further, by examining daily networks, we demonstrated that diverse rewarding plants for the effective pollinator provided an ecological context for effective pollination of the deceptive orchid, which was postulated by previous researchers [26,27]. We cannot conclude whether the higher nestedness in the orchid network necessarily enabled the orchid to be pollinated due to the low sampling effort in the network survey. However, given that nestedness increases with network complexity (i.e., the number of interactions) [33], the results of this study, at least, suggest that high plant and pollinator richness can increase the chance that the deceptive orchid will be pollinated.

Network metrics can provide important insight into species conservation, in terms of community persistence [33,36,37]. However, given the limited resources, including cost, labor, and time needed for managing the conservation projects of endangered species, it may be difficult to allocate the majority of the resources to a network survey that requires high sampling intensity [34]. In the present study, the network metrics (nestedness, specialization, and modularity) calculated in a single habitat may be inaccurate due to the short survey time (4 d). Low sampling effort can lead to the misinterpretation of network metrics, even though the null model for the network was used [51,52]. Instead, comparing the metrics between the networks can lend meaningful insight into the ecological factors affecting the network properties, as in the study by Song et al. [40]. When employing translocation measures, finding a habitat that is ecologically suitable is the most important. Especially a deceptive orchid, such as that of *C. guttatum,* needs a specific ecological context for successful pollination, as shown in the daily network patterns in the present study (Figure 6 and Table 6). In this regard, if *C. guttatum* is translocated beyond its original habitat, investigating the plant–pollinator network in several experimental sites and comparing the metrics among the networks for each site will provide novel insight into understanding specific network structures that enable the orchid to be pollinated.

Although the foraging spectrum of the pollinators visiting *Cypripedium* species is generally diverse [20,21,22,23,24,25], pollinators visiting *C. guttatum* may have a weak color preference for flowers. *C. guttatum,* as a food-deceptive orchid, is pollinated by three sweat bee species (e.g., *Lasioglossum virideglaucum*, *L*. *clypeinitens*, and *L*. *sauterum*) in China [21]. In this study, *C. guttatum* was only pollinated by *L*. *virideglaucum*, and the other species (e.g., *L*. *clypeinitens*, and *L*. *sauterum*) present in China were not observed. This may be due to the low diversity of sweat bees that fit into the posterior opening size of the flower or the low number of *C. guttatum* individuals (i.e., four individuals). However, given that *L*. *virideglaucum* consistently pollinated *C. guttatum* flowers over two countries (i.e., Korea and China), the pollinator is likely to have a preference for the specific colors of both the orchid and co-occurring flowers. Bänziger et al. [21] reported that *L*. *virideglaucum* visited co-occurring flowers with white, purple, and yellow colors, which is consistent with our results in the *C. guttatum* module (Figure 5). The color preference of the sweat bee can be explicitly tested using artificial flowers that have the same shape and color as the flowers of *C. guttatum* in a future study.

Our study may provide some useful insight into translocation actions for *C. guttatum* in the Republic of Korea. For example, *A*. *gemmifera* was determined to be a keystone species that can provide essential pollen and nectar for *L*. *virideglaucum*. These findings revealed that such a key species supporting the orchid pollinator could be identified by a network study, as reported by Phillips et al. [28]. Thus, when selecting a suitable habitat for the survival of *C. guttatum*, whether two keystone species (e.g., *A*. *gemmifera* and *L*. *virideglaucum*), which highly contribute to the pollination of the orchid, are present should be considered first. Given that the effective pollinator *L*. *virideglaucum* is only distributed in Gangwon Province and Hallasan in Jeju Island in Korea [53], and the distribution of *C. guttatum* is restricted above 1000 m a.s.l. or at relatively high latitudes worldwide (Bänziger et al. [21] and this study), one should consider Gangwon and Jeju, around 1000 m a.s.l., as suitable habitats for the reintroduction or assisted colonization of the orchid. Overall, despite the short term of this survey, the results suggest that network analysis can be a useful conservation tool when investigating suitable habitats for food-deceptive orchids, by finding an ecological context in which the orchids can be pollinated.

## Figures and Tables

**Table 1:** List of insect-pollinated plants flowering at the Manhangjae site in the Republic of Korea during the survey period (12 May to 7 June 2018).

No.	Family Name	Scientific Name
1	Liliaceae	*Polygonatum odoratum* var. *pluriflorum*
2	Ranunculaceae	*Ranunculus japonicus*
3	Violaceae	*Viola mandshurica*
4	Ericaceae	*Rhododendron schlippenbachii*
5	Orchidaceae	*Cypripedium guttatum*
6	Rosaceae	*Aruncus dioicus* var. *kamtschaticus*
7	Caprifoliaceae	*Weigela florida*
8	Brassicaceae	*Arabis gemmifera*
9	Asteraceae	*Taraxacum officinale*
10	Brassicaceae	*Barbarea vulgaris*
11	Valerianaceae	*Valeriana fauriei*
12	Caryophyllaceae	*Cerastium holosteoides* var. *hallaisanensis*

**Table 2:** Visitation duration of the effective pollinator *Lasioglossum virideglaucum* on *Cypripedium guttatum*.

No.	Entering Time	Escaping Time	Visitation Duration (s)
1	13:53:09	13:54:05	55.0
2	14:35:37	14:36:45	68.0
3	14:37:40	14:38:40	60.0
4	14:38:42	14:40:00	78.0
5	12:58:55	13:00:02	67.0
6	12:50:05	12:51:16	71.0
7	13:59:05	14:00:02	57.0
8	14:20:40	14:21:55	45.0
Mean			62.0
Standard deviation			10.4

**Table 3:** Visitation frequency of the effective pollinator *Lasioglossum virideglaucum* to four flowering shoots of *Cypripedium guttatum*.

Date	Time of Day						
	0900–1000 h	1000–1100 h	1100–1200 h	1200–1300 h	1300–1400 h	1400–1500 h	1500–1600 h
28 May 2018	0	2	2	2	1	3	0
31 May 2018	0	0	2	1	2	3	0
4 June 2018	0	0	0	0	0	0	0

**Table 4:** Unweighted degree and centrality scores (BC: betweenness centrality, CC: closeness centrality) for pollinators that occurred in the *Cypripedium guttatum* habitat (the Manhangjae site) in the Republic of Korea.

Higher Trophic Level (i.e., Pollinator)	Unweighted Degree	Betweenness Centrality	Closeness Centrality
*Eucera* sp. 1	1	0.000	0.030
*Pristomyrmex pungens*	1	0.000	0.055
*Ceratina flavipes*	1	0.000	0.055
*Lasioglossum exiliceps*	2	0.016	0.057
*Lasioglossum virideglaucum*	8	0.259	0.070
*Lasioglossum duplex*	3	0.083	0.062
*Lasioglossum miyabei*	2	0.153	0.047
*Eristalomyia tenax*	1	0.000	0.049
*Metasyrphus luniger*	5	0.176	0.068
*Curculionidae* sp. 1	2	0.016	0.053
*Oedemeridae* sp. 1	3	0.188	0.063
*Elateridae* sp. 1	1	0.000	0.035
*Vespidae* sp 1	1	0.000	0.044
*Parnassius stubbendorfii*	2	0.035	0.061
*Bombylius major*	2	0.024	0.057
*Carterocephalus silvicola*	1	0.000	0.049
*Andrena* sp 1	2	0.005	0.047
*Apis mellifera*	3	0.044	0.059
*Philopota nigroaenea*	1	0.000	0.042

**Table 5:** Unweighted degree and centrality scores (BC, betweenness centrality; CC, closeness centrality) of plants found in the *Cypripedium guttatum* habitat (the Manhangjae site) in the Republic of Korea.

Lower Trophic Level (i.e., Plant)	Unweighted Degree	Betweenness Centrality	Closeness Centrality
*Polygonatum odoratum* var. *pluriflorum*	2	0.000	0.059
*Ranunculus japonicus*	10	0.238	0.106
*Viola mandshurica*	0	-	-
*Rhododendron schlippenbachii*	5	0.048	0.100
*Cypripedium guttatum*	1	0.000	0.095
*Aruncus dioicus* var. *kamtschaticus*	2	0.000	0.065
*Weigela florida*	4	0.000	0.095
*Arabis gemmifera*	5	0.476	0.106
*Taraxacum officinale*	1	0.000	0.078
*Barbarea vulgaris*	1	0.000	0.095
*Valeriana fauriei*	7	0.238	0.106
*Cerastium holosteoides* var. *hallaisanensis*	4	0.000	0.095

**Table 6:** Parameters and combined nestedness (NODF_c_) of the daily networks. Raw NODF is the raw nestedness value, and max NODF is the maximum nestedness of a network with the same number of species and links as the focal network, which can be calculated only when every species has at least one link. The max NODF values for days 147, 149, and 154 were calculated by adding 3, 3, and 1 link(s) to their original matrices to satisfy the analysis requirement (i.e., the total number of plant and animal species ≤ number of links). In this case, the Max NODF will not be accurate, but the estimate is conservative.

	Survey Date (Julian)			
	146	147	149	154
Network size (plants × animals)	8 × 14	4 × 3	10 × 9	4 × 8
Total number of links (binary)	25	5	17	12
Raw NODF	0.239	0.333	0.216	0.353
Max NODF	0.598	0.889	0.469	0.647
NODF_c_	1.571	1.668	2.495	1.933

## Data Availability

The data are available from the authors upon request.

## Abbreviations

Confidence interval (CI), nestedness based on overlap and decreasing fill (NODF), combined nestedness (NODF_c_), betweenness centrality (BC), closeness centrality (CC).
